# Assessment of Preoxygenation using Real-Time End-Tidal Oxygen Measurements Versus Single-Breath End-Tidal Oxygen Measurements in Healthy Volunteers

**DOI:** 10.1016/j.acepjo.2025.100079

**Published:** 2025-02-28

**Authors:** Steven Lindsey, Tim P. Moran, John Diehl, James Snitzer, Kellie L. McKenzie, Nabeel Janjua, Jeremy Ackerman, Alayna McLaughlin, Rachel MacAskill, Stephen M. Carroll

**Affiliations:** 1 Department of Emergency Medicine, Emory University School of Medicine, Emory University, Atlanta, Georgia, USA; 2 Christiana Care Department of Emergency Medicine, Newark, Delaware, USA

**Keywords:** endotracheal intubation, preoxygenation, pre-oxygenation, end-tidal oxygen, supplemental oxygen

## Abstract

**Objective:**

Preoxygenation decreases morbidity for patients requiring endotracheal intubation. However, rigorous means for determining adequate pre-oxygenation are limited in the emergency department (ED). End-tidal oxygenation (EtO_2_) monitoring could potentially improve preoxygenation in the ED. The accuracy of nasal cannula (NC) EtO_2_ in patients receiving supplemental oxygen is unknown. Our study examined the correlation between NC EtO_2_ and single-breath (SB) EtO_2_.

**Methods:**

Healthy volunteers were randomized to receive supplemental oxygen via a nonrebreather mask (NRBM) or a noninvasive ventilation mask (NIV). Participants underwent 3-minute trials at 3 different settings: NRBM at 15 liters per minute (LPM), 35 LPM, and 55 LPM, or NIV at 40% fraction of inspired oxygen (FiO_2_), 70% FiO_2_, and 100% FiO_2_. NC EtO_2_ and SB EtO_2_ were obtained at the end of each trial.

**Results:**

Complete data were obtained for 104 participants. Beta regression analysis revealed a strong correlation between NC EtO_2_ and SB EtO_2_ in the NIV group (pR^2^ = 0.7) and a moderate correlation (pR^2^ = 0.4) in the NRBM group. Mean differences in the NRBM arm were 13.1% (15 LPM), 18.0% (35 LPM), 17.1% (55 LPM), and 1.8% (40% FiO_2_), 5.1% (70% FiO_2_), and 10.1% (100% FiO_2_) in the NIV arm.

**Conclusions:**

Supplemental oxygen led to an overestimation of NC EtO_2_ across both groups, with NRBM more than NIV. The correlation between SB EtO_2_ and NC EtO_2_ suggests NC EtO_2_ may be useful in assessing preoxygenation in real-time. Further study is needed to examine its clinical efficacy in preventing desaturation events during endotracheal intubation in the ED.

**The Bottom Line**

End-tidal oxygen monitoring has the potential to better assess the preoxygenation of patients before intubation and, therefore, minimize risk to our patients. This study aimed to determine whether supplemental oxygen delivery would affect end-tidal oxygen measurements in healthy volunteers. In general, the addition of supplemental oxygen resulted in falsely elevated end-tidal oxygen readings in the range of 2% to 18%, depending on the oxygen delivery method. Our findings will help to better inform preintubation decisions, as emergency department physicians can better estimate the patient’s true end-tidal oxygen levels while pre-oxygenation is in progress.

## 1 Introduction

### 1.1 Background

Preoxygenation is a well-established practice that denitrogenates the lungs and prolongs safe apnea time during endotracheal intubation,^1^ as hypoxia during intubation can lead to adverse patient outcomes.^2^, ^3^, ^4^, ^5^ In the emergency department (ED), 3 minutes of full tidal breaths with high flows of oxygen via either a nonrebreather mask (NRBM) or noninvasive ventilation mask (NIV)^6^^,^^7^ is generally used as a surrogate for adequate preoxygenation.^8^, ^9^, ^10^, ^11^ Pulse oximetry readings can also be used as an adjunct for the determination of adequate preoxygenation; however, this device does not directly measure the percentage of oxygen present in the lungs.^10^

### 1.2 Importance

In-line end-tidal oxygen (EtO_2_) measurements have been well studied in operating room settings and directly correlate with the prevention of oxygen desaturation events when the EtO_2_ (the percentage of oxygen in an exhaled breath) is greater than 90% before the induction of apnea.^1^^,^^10^^,^^12^^,^^13^ In contrast to the operating room setting, the use of bag-valve masks to preoxygenate patients is generally avoided in the ED setting,^13^ limiting the feasibility of in-line EtO_2_ detection. Single-breath (SB) EtO_2_ measurement is the gold standard for preoxygenation and has been well-validated.^7^^,^^14^, ^15^, ^16^ Unfortunately, this method is also impractical in patients with respiratory distress. The most pragmatic EtO_2_ monitoring method in the ED setting is via real-time oxygenation sensors attached to a nasal cannula (NC); however, limited research exists examining the accuracy of real-time EtO_2_ measurements in patients receiving supplemental oxygen.

### 1.3 Goals of This Investigation

We hypothesized that the supplemental oxygen flowing through the EtO_2_ sensor would falsely elevate the readings, but that this error created by supplemental oxygen could be quantified. Our primary outcome was to estimate the concordance of EtO_2_ between real-time NC and SB in healthy volunteers receiving supplemental oxygen for 3 minutes. Our secondary outcome was to evaluate the difference between SB EtO_2_ at the time when the NC device reached maximum EtO_2_ (Tmax_NC_) compared with SB EtO_2_ at 3 minutes.

## 2 Methods

### 2.1 Study Setting and Selection of Subjects

Volunteers were recruited from the Grady Memorial Hospital staff, the Emory University School of Medicine, and the Morehouse School of Medicine and enrolled between September 2019 and October 2023. Enrollment was paused from February 2020 until July 2023 due to the COVID-19 pandemic. A total of 105 participants began the study, with 1 withdrawing prior to any data collection due to discomfort from NIV. The study was approved by our institutional review board (IRB) and registered at ClinicalTrials.gov (NCT03840486). We obtained written consent from all participants and allowed them to withdraw from the study at any time.

Inclusion criteria included the following: age >18 years, self-identified as being in good health, grossly normal dentition as determined by study investigators, no self-reported symptoms of upper respiratory infection or other infectious process, no history of severe pulmonary disease or asthma requiring daily use of an inhaler, and for female participants, self-report of not being pregnant. Study participants were excluded if they did not agree to study enrollment or were unable to tolerate the entire course of supplemental oxygen required to complete the study.

### 2.2 Study Design and Interventions

**Figure 1:**
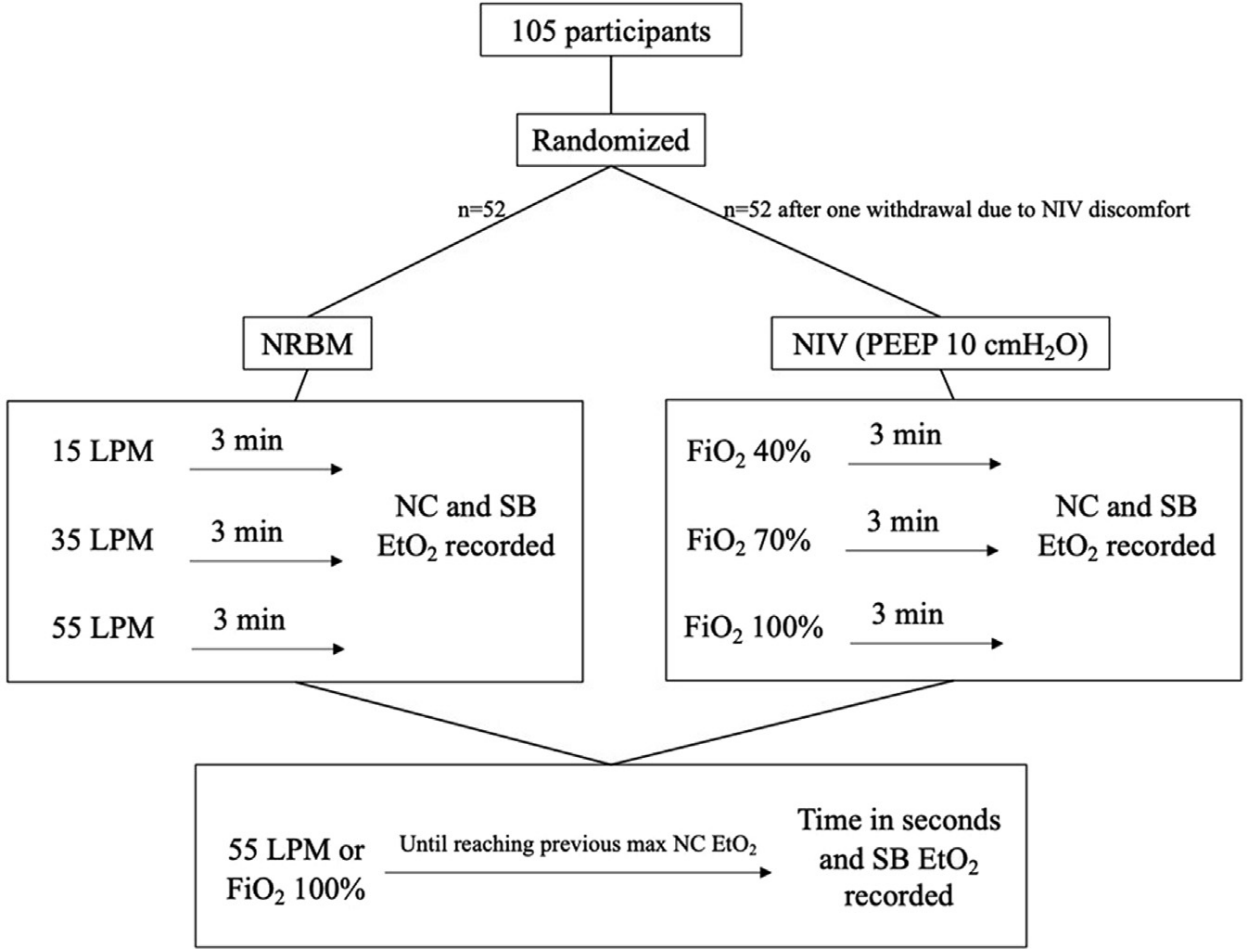
Study flow diagram for the real-time end-tidal oxygen (ETO_2_) measurement study. FiO_2_, fraction of inspired oxygen; LPM, liters per minute; NC, nasal cannula; NIV, noninvasive ventilation mask; NRBM, nonrebreather mask; PEEP, positive end-expiratory pressure; SB, single-breath.

Following screening and informed consent, participants were randomized to either the (1) NRBM or (2) NIV arms of the study, along with providing study investigators with their self-reported age, gender, height, and weight (Fig 1). The NC device (NomoLine LH Adult Nasal/Oral CO_2_ Cannula; Masimo) was placed on each participant, and investigators confirmed adequate morphology of EtO_2_ waveforms on the gas analyzer monitor (ISA OR+ module and Root monitor). SB EtO_2_ measurements were obtained using a gas analyzer (Handi+, model R218P12; Maxtec) attached to a piece of standard corrugated ventilator tubing.

Before delivering supplemental oxygen, an SB EtO_2_ measurement was obtained to establish a baseline. Then, the NRBM (1059 adult nonrebreather mask with safety vent; Hudson RCI) or NIV mask (AF531 Oro-Nasal Mask, size medium on V60 ventilator; Philips Respironics) was placed over the NC device. Each subject participated in 3 3-minute trials of variable oxygen delivery. For the NRBM arm, this entailed trials at 15 LPM, 35 LPM, and 55 LPM. In the NIV arm, trials consisted of a 40% fraction of inspired oxygen (FiO_2)_, 70% FiO_2_, and 100% FiO_2_, all at positive end-expiratory pressure = 10 cm H_2_0. For the NRBM study arm, an oxygen flow meter capable of measuring up to 70 LPM (Oxygen Chrome Flowmeter 1MFA8001EC; Precision Medical) was used to achieve accurate flow rates. Study participants were randomized regarding the order in which they received the varied flow and FiO_2_ rates.

After each trial, study investigators noted the NC EtO_2_ reading and requested that the study participants hold their breath. With the assistance of the study investigators, the study participants removed the NRBM or NIV mask and breathed into the SB EtO_2_ gas analyzer. At the end of each trial and following several minutes of breathing room air, SB EtO_2_ readings were obtained to ensure that the study participant’s SB EtO_2_ readings returned to their room air baseline. This process was repeated twice at a different LPM or FiO_2_ (depending on group allocation).

To determine the time required to reach the maximum EtO_2_ on the NC device (Tmax_NC_), a fourth trial was performed at 55 LPM or 100% FiO_2_ (depending on group allocation), following the NC level continuously until it reached its previous maximum. We then recorded the time in seconds and measured an SB EtO_2_ at that time (Tmax_NC_), allowing for a comparison of SB EtO_2_ at Tmax_NC_ versus SB EtO_2_ at a time of 3 minutes.

### 2.3 Outcomes

The primary outcome was the degree of oxygenation measured at each flow rate. The study was sufficiently powered to detect a 5% difference in EtO2 between the NC and SB measurements. Five percent was chosen because it equates to approximately 30 extra seconds of safe apnea time in an 80-kg male (5% × 2400 mL/O_2_ consumption at 250 mL/min). Based on previous studies with similar designs, we estimated that the standard deviation of EtO2 would be approximately 6.8%.^7^^,^^15^ These values were used to estimate the sample size required to achieve 80% power at an alpha of 0.0085 (Sidak-corrected *P* value for 6 tests). This analysis returned a total sample size of 104 (52 per arm).^10^ The secondary outcome was the time taken to reach the maximum EtO_2_ measurements.

### 2.4 Data Analysis

Categorical variables were described using frequencies and percentages. Continuous/scale variables were described using medians with interquartile ranges and means with standard deviations. The degree of association between NC EtO_2_ and SB EtO_2_ across the oxygen delivery levels was assessed in 2 ways. First, because EtO_2_ is a percentage, this was evaluated using mixed-effects regression with a beta distribution and logit link. The mixed-effects model was used to account for multiple measurements from the same participant. To assess the degree of association, we present (1) the pseudo-R^2^ (ie, the square of the correlation between the model-predicted and observed values) and (2) the root mean squared error (RMSE; ie, the square root of the mean of the squared errors). Second, we computed the paired sample mean difference and 95% confidence interval for that difference between SB EtO_2_ and NC EtO_2_. Confidence intervals were computed using bias-corrected and accelerated bootstrap resampling (100,000 resamples were used to generate stable estimates). For our secondary outcome, we compared the NIV and NRBM groups for the time taken (Tmax_NC_) to reach the maximum EtO_2_ measurements. Mean and median times were estimated using the Kaplan-Meier method. Kaplan-Meier curves were compared using the log-rank test. Analyses were conducted using R (v. 4.3.2; R Core Team, 2023). SB EtO_2_ at Tmax_NC_ and SB EtO2 at 3 minutes were compared by calculating the paired sample mean difference and 95% confidence interval for that difference.

## 3 Results

A total of 104 participants completed the study. The majority identified as female (64.4%) and the median age was 28 (IQR, 26-33.5) years. The median baseline body mass index was 24.8 (IQR, 21.9-28.3) and the median SB baseline EtO_2_ was 16.8% (IQR, 16.1-17.5). Table 1 presents the baseline characteristics stratified by the study arm.

**Table 1:** Baseline characteristics of study participants in the real-time end-tidal oxygen (ETO_2_) measurement study, September 2019-October 2023.

Characteristic	NIV	NRBM	Total
Age (y), median (IQR)	28 (26-34)	27 (25-34.5)	28 (26-33.5)
Gender, n (%)			
Female	30 (57.7)	37 (71.2)	67 (64.4)
Male	22 (42.3)	15 (29.8)	37 (35.6)
BMI (kg/m^2^), median (IQR)	24.7 (23-28.2)	24.9 (21.5-28.3)	24.8 (21.9-28.3)
Baseline EtO_2_, median (IQR)	16.8% (16.1%-17.3%)	16.8% (16%-17.8%)	16.8% (16.1%-17.5%)

BMI, body mass index; IQR, interquartile range NIV, noninvasive ventilation; NRBM, nonrebreather mask.

**Figure 2:**
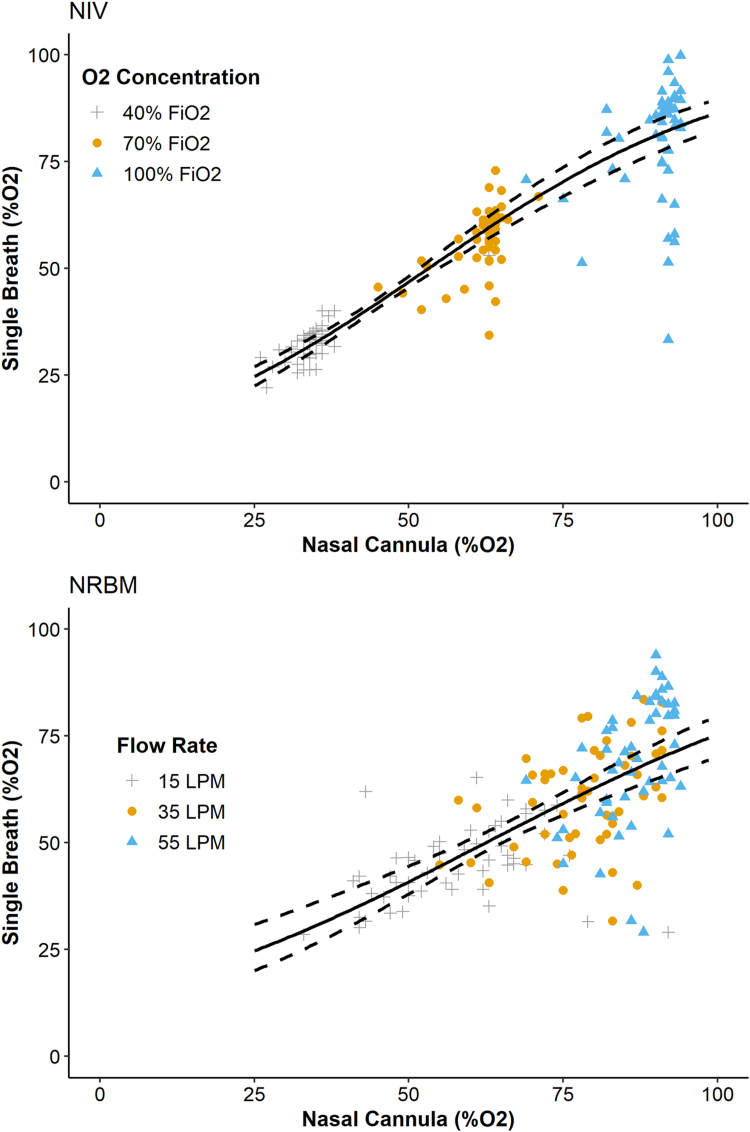
Scatterplot describing the association between real-time end-tidal oxygen nasal cannula measurements and single-breath end-tidal oxygen measurements in the noninvasive ventilation (top) and nonrebreather mask (bottom) study arms. The solid black line depicts the line of best fit from the beta regression; the dashed lines depict the 95% confidence interval for the line of best fit. FiO_2_, fraction of inspired oxygen; LPM, liters per minute; NIV, Noninvasive ventilation mask; NRBM, Nonrebreather mask.

EtO_2_ measurements between NC and SB were significantly correlated in both arms of the study, although this correlation was considerably stronger in the NIV arm (Fig 2, Table 2). For NIV participants, the pseudo-R^2^ was 0.72 (95% CI, 0.56-0.83), and the model RMSE indicated that the predicted SB value differed from the observed SB value by an average of approximately 8.5% EtO_2_. For the NRBM arm, the pseudo-R^2^ was 0.43 (95% CI, 0.30-0.55), and the model RMSE indicated that the predicted SB value differed from the observed SB value by an average of approximately 11.7% EtO_2_. The formulas for determining SB EtO_2_ based on the NC EtO_2_ readings for the NIV and NRBM groups can be found in Supplementary Appendix 1.

**Table 2:** Regression table describing the association between real-time end-tidal oxygen nasal cannula measurements and single-breath end-tidal oxygen measurements in the noninvasive ventilation (NIV) and nonrebreather mask (NRBM) study arms.

Group	pR^2^ (95% CI)	r	RMSE	*P*
NIV	0.72 (0.56-0.83)	0.85	8.46	< .001
NRBM	0.43 (0.30-0.55)	0.66	11.68	< .001

CI, confidence interval; pR^2^, pseudo R-squared; r, linear correlation; RMSE, root-mean-square error.

**Figure 3:**
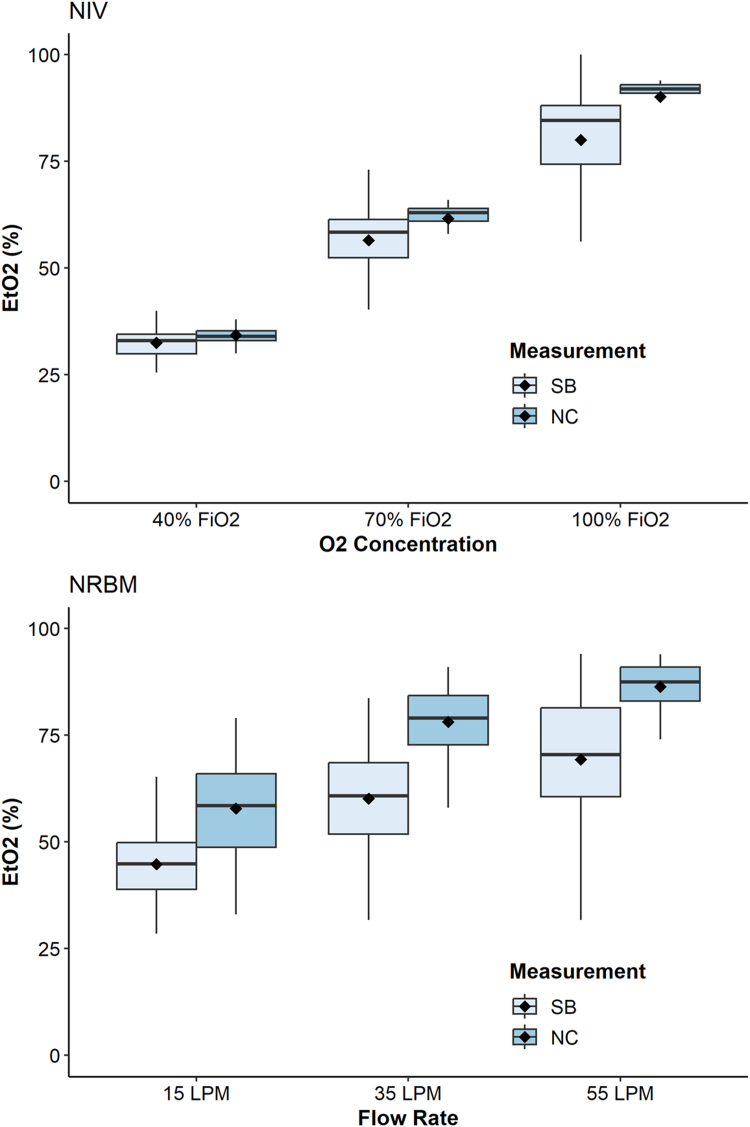
Mean absolute differences between the single-breath (SB) end-tidal oxygen measurements (ETO_2_) and real-time nasal cannula (NC) ETO_2_ stratified by flow rates/oxygen concentrations for the noninvasive ventilation (NIV, top) and nonrebreather mask (NRBM, bottom) groups. The bottom and top of the boxes depict the 25th and 75th percentiles, respectively. The line contained within the boxes depicts the median. The diamonds depict the mean. FiO_2_, fraction of inspired oxygen; LPM, liters per minute.

The mean differences between the NC EtO_2_ and SB EtO_2_ measurements following 3 minutes in the NIV group were 1.8% (1.1%-2.7%), 5.1% (3.5%-7.1%), and 10.0% (7.3%-14.3%), at 40% FiO_2_, 70% FiO_2_, and 100% FiO_2_, respectively (Fig 3 top and Table 3). In the NRBM arm, mean differences between NC EtO_2_ and SB EtO_2_ at 3 minutes were 13.1% (10.3%-16.8%) at 15 LPM, 18.0% (14.9%-21.4%) at 35 LPM, and 17.1% (13.9%-21.0%) at 55 LPM (Fig 3 bottom and Table 3). All 6 comparisons were statistically significant (*P* < .0085).

**Table 3:** Nasal cannula (NC) real-time end-tidal oxygen (ETO_2_) measurements and single-breath (SB) ETO_2_ measurements across increasing oxygen concentrations and flow rates for the noninvasive ventilation (NIV) and nonrebreather mask (NRBM) study arms stratified by flow rate/concentration and study arm.

Parameters	NC	SB	Difference (95% CI)
NIV			
40% FiO_2_			
Mean (±SD)	34.3% (4.8)	32.5% (4.7)	1.8% (1.1-2.7)
Median (IQR)	34% (33- 35.8)	33.1% (29.9-34.6)	
70% FiO_2_			
Mean (±SD)	61.6% (4.4)	56.6% (7.7)	5.1% (3.5-7.1)
Median (IQR)	63% (6164)	58.4% (52.2-61.5)	
100% FiO_2_			
Mean (±SD)	90.1% (5.0)	80.1% (13.3)	10.0% (7.3-14.3)
Median (IQR)	92% (91-93)	84.6% (73.7-88.1)	
NRBM			
15 LPM			
Mean (±SD)	57.8% (11.6)	44.8% (6.9)	13.1% (10.3-16.8)
Median (IQR)	58.5% (48.3-66)	44.9% (38.7-50.1)	
35 LPM			
Mean (±SD)	78.2% (8.9)	60.2% (12.2)	18.0% (14.9-21.4)
Median (IQR)	79% (72.3-84.8)	60.8% (51.4-69.4)	
55 LPM			
Mean (±SD)	86.4% (5.7)	69.3% (14.8)	17.1% (13.9-21.0)
Median (IQR)	87.5% (83-91)	70.5% (60.3-82.1)	

FiO_2_, fraction of inspired oxygen; IQR, interquartile range; LPM, liters per minute; SD, standard deviation.

The mean time to reach a maximum reading on NC EtO_2_ (Tmax_NC_) was 130 seconds (118 - 140 seconds) in the NIV arm and 116 seconds (95-138 seconds) in the NRBM arm (Table 4). There was no significant difference between the two groups (*P* = .14). The mean difference between SB EtO_2_ at Tmax_NC_ and SB EtO_2_ at 3 minutes was −1.5% (−4.7% to 1.7%) and 1.1% (−2.0% to 4.6%) in the NIV and NRBM groups, respectively. Appendix 2 contains the Kaplan-Meier curves depicting the time it takes to reach Tmax_NC_.

**Table 4:** Time required to reach the 3-minute nasal cannula (NC) end-tidal oxygen (EtO_2_) reading and the mean difference between the single-breath (SB) EtO_2_ measurement at Tmax_NC_ and 3 minutes for the noninvasive ventilation and nonrebreather mask arms.

Parameter	Mean time to Tmax_NC_ (95% CI)	Median time to Tmax_NC_ (95% CI)	SB EtO_2_ at 3 minutes (95% CI)	SB EtO_2_ at Tmax_NC_ (95% CI)	Mean Difference (95% CI)
NIV	129.8 s (117.5-142)	126 (107.2-144.8)	80.1% (75.9-83.2)	78.5% (75.0-81.7)	−1.5% (−4.7-1.7)
NRBM	116.2 s (94.7-137.4)	100 (88.5-111.5)	69.3% (65.1-73.1)	70.4% (66.3-73.8)	1.1% (−2.0-4.6)

NIV, noninvasive ventilation study arm; NRBM, nonrebreather mask study arm.

## 4 Limitations

This study was limited to healthy volunteers. While this inclusion criterion allowed for safely evaluating the correlation between NC EtO_2_ and SB EtO_2_, this study population does not reflect the target population of those in the ED who are critically ill and who often have underlying lung pathology and inadequate ventilation. This correlation would be less reliable in patients with hypoventilation, as the NC sensor might theoretically report a higher EtO_2_ reading due to a higher ratio of ambient supplemental oxygen to expired gas. It is also possible critically ill patients with hypoventilation could be found to have falsely decreased time to first reach maximum NC EtO_2_ for the same reason.^13^^,^^17^ Additionally, in having our participants hold their breath before breathing into the SB sensor, it is possible that the larger last breath may have spuriously elevated the EtO_2_ reading. From a practical standpoint, this is unavoidable and reflects the methodology of other, similar studies.^15^^,^^18^ To address these limitations, future directions would include evaluating real-time NC ETO_2_ measurements in a large, randomized controlled trial enrolling ED patients presenting with respiratory failure.

## 5 Discussion

In our study of healthy volunteers, we compared the NC EtO_2_ device with the gold standard SB EtO_2_ device in participants receiving NIV at different FiO_2_ levels and NRBM at different flow rates and saw a significant correlation between NC EtO_2_ and SB EtO_2_ in each arm at 3 minutes. In general, increasing O_2_ delivery introduced more EtO_2_ discordance between the devices.

Previous studies have demonstrated that hypoxic events are common during ED ETI^19^ and higher EtO_2_ readings directly correlate with longer safe apnea times,^20^ yet EtO_2_ is rarely used in the ED setting.^21^ In the most practical application of EtO_2_ tracking in the ED setting to date, Oliver et al^22^ determined that monitoring EtO_2_ periintubation significantly increased the quality of preoxygenation, with 67% of monitored patients reaching an EtO_2_ > 85% compared with 26% without monitoring.^18^ This was associated with a decrease in hypoxic events from 18% to 8%. One limitation of this study, as pointed out by the authors, is that they did not account for the potential error created by the supplemental oxygen being delivered, and a smaller proportion of patients may be reaching an EtO_2_ > 85%. Our study addresses this gap by quantifying the effects of supplemental oxygen blowing by the device to better inform the appropriate implementation of NC EtO_2_ monitoring in the ED.

For our primary outcome, we set an a priori mean difference of 5% between SB EtO2 and NC EtO2 as a surrogate for 30 seconds of apnea time. Most of the NC EtO_2_ readings were not within our clinically significant 5% of SB EtO2 readings but demonstrate the better performance of the NC EtO_2_ device at lower O_2_ delivery levels (ie, 15 LPM and 40% FiO_2_). These data confirm our hypothesis that a portion of administered oxygen was detected using the NC EtO_2_ sensor across all groups, particularly in the NRBM arm. This may potentially confound the clinician’s ability to discern the patient’s actual (ie, SB) EtO_2_. Based on the mean differences between NC EtO_2_ and SB EtO_2_ in our study, one would expect the SB EtO_2_ to be, on average, 13% to 17% lower than that displayed on the NC EtO_2_ reading in a healthy person on an NRBM and, on average, 2% to 10% lower in a healthy person breathing NIV. This result is to be expected as the NIV system is a closed circuit (except for an intentional air leak built into the system), whereas the NRBM is a semi-open system that allows active entrainment of room air, which would be expected to affect the real-time EtO_2_ readings.

While the mean differences above provide a quick estimation of the patient’s true EtO_2_ at the bedside, the correlations between the NC and SB devices allow for a more accurate representation of the patient’s EtO_2_ (Supplementary Appendix 1). Our data show that, in healthy volunteers, EtO_2_ estimated from the NC device is highly correlated with actual SB EtO_2_ in the NIV group and moderately correlated with actual SB EtO_2_ in the NRBM group. These findings suggest that NC EtO_2_ can be used as a proxy for SB EtO_2_ when the latter is not available.

The EtO_2_ values obtained in our study were lower than those seen in previous studies evaluating preoxygenation with various devices. Specifically, our mean SB EtO_2_ reading for NRBM at 15 LPM was 44.8% at 3 minutes, which is lower than the 52% to 74% range as reported in other studies.^7^^,^^14^, ^15^, ^16^^,^^23^ This may have been related to increased mask leak/entrainment of room air at this lower flow rate. In contrast, the mean SB EtO_2_ of 69.3% at 55 LPM was closer to the 74% to 86% seen in other studies, suggesting that the additional flow limited the mixing of room air.^7^^,^^24^ The only other comparable study for NIV found similar SB EtO_2_ readings as ours at 100% and positive end-expiratory pressure of 10 cc H_2_0, (78.6% and 80.1%, respectively).^25^ These similarities at higher flow rates between our study and others increase the reliability of our findings.

For our secondary outcome, the time required to reach maximum NC EtO_2_ was shorter than the traditionally accepted 3 minutes required for adequate preoxygenation,^10^ and the accuracy of this was confirmed using SB EtO_2_ readings. The small, nonsignificant differences observed between SB EtO_2_ at Tmax_NC_ and 3 minutes suggest that the supplemental oxygen was not “overwhelming” the NC EtO_2_ device, ie, displaying an EtO_2_ value higher than was in the lungs, at the earlier time point. In the clinical setting, this may translate into a simple method for earlier detection of adequate preoxygenation and therefore an earlier time to safely initiate endotracheal intubation.

Our study demonstrated that estimates of a healthy volunteer’s EtO_2_ via an NC sensor can be made with a reasonable degree of accuracy, even in the setting of supplemental oxygen delivery. This real-time data could allow improved decision-making during ED endotracheal intubation by more accurately identifying the preoxygenation plateau and more reliably estimating preoxygenation adequacy. This could result in a quicker decision to proceed with intubation if EtO_2_ measurements are deemed sufficient. Conversely, if EtO_2_ measurements are not considered adequate, different ETI techniques other than rapid sequence intubation (such as delayed sequence intubation or awake intubation) or different preoxygenation strategies can be employed. This knowledge of the patient’s preoxygenation status could have a direct effect on decreasing severe desaturations during ED endotracheal intubation.

## Author Contributions

SC and TM conceived the study, designed the protocol, and obtained research funding. SL and SC supervised the conduct of the trial and data collection. SL, KM, NJ, JD, JA, AM, RM, JS, and SC undertook the recruitment of participating centers and patients and managed the data. TM provided statistical advice on study design and analyzed the data. SL drafted the manuscript and all authors contributed substantially to its revision. SL and SC take responsibility for the paper in its entirety.

## Funding and Support

This study was funded by the 10.13039/100012182Emory Medical Care Foundation internal grant.

## Conflict of Interest

The authors declare that they have no known competing financial interests or personal relationships that could have appeared to influence the work reported in this paper.

## References

[1] Frerk C., Mitchell V.S., McNarry A.F. (2015). Difficult Airway Society 2015 guidelines for management of unanticipated difficult intubation in adults. Br J Anaesth.

[2] Russotto V., Rahmani L.S., Parotto M., Bellani G., Laffey J.G. (2022). Tracheal intubation in the critically ill patient. Eur J Anaesthesiol.

[3] Davis D.P., Dunford J.V., Poste J.C. (2004). The impact of hypoxia and hyperventilation on outcome after paramedic rapid sequence intubation of severely head-injured patients. J Trauma.

[4] De Jong A., Rolle A., Molinari N. (2018). Cardiac arrest and mortality related to intubation procedure in critically Ill adult patients: A multicenter cohort study. Crit Care Med.

[5] Heffner A.C., Swords D.S., Neale M.N., Jones A.E. (2013). Incidence and factors associated with cardiac arrest complicating emergency airway management. Resuscitation.

[6] Popowicz P., Leonard K. (2022). Noninvasive ventilation and oxygenation strategies. Surg Clin North Am.

[7] Driver B.E., Prekker M.E., Kornas R.L., Cales E.K., Reardon R.F. (2017). Flush rate oxygen for emergency airway preoxygenation. Ann Emerg Med.

[8] Berthoud M., Read D.H., Norman J. (1983). Pre-oxygenation--how long?. Anaesthesia.

[9] Pandit J.J., Duncan T., Robbins P.A. (2003). Total oxygen uptake with two maximal breathing techniques and the tidal volume breathing technique: A physiologic study of preoxygenation. Anesthesiology.

[10] Tanoubi I., Drolet P., Donati F. (2009). Optimizing preoxygenation in adults. Can J Anaesth.

[11] Weingart S.D., Levitan R.M. (2012). Preoxygenation and prevention of desaturation during emergency airway management. Ann Emerg Med.

[12] Sajayan A., Wicker J., Ungureanu N., Mendonca C., Kimani P.K. (2016). Current practice of rapid sequence induction of anaesthesia in the UK - a national survey. Br J Anaesth.

[13] Nimmagadda U., Salem M.R., Crystal G.J. (2017). Preoxygenation: Physiologic basis, benefits, and potential risks. Anesth Analg.

[14] Groombridge C., Chin C.W., Hanrahan B., Holdgate A. (2016). Assessment of common preoxygenation strategies outside of the operating room environment. Acad Emerg Med.

[15] Hayes-Bradley C., Lewis A., Burns B., Miller M. (2016). Efficacy of nasal cannula oxygen as a preoxygenation adjunct in emergency airway management. Ann Emerg Med.

[16] Groombridge C.J., Ley E., Miller M., Konig T. (2017). A prospective, randomised trial of pre-oxygenation strategies available in the pre-hospital environment. Anaesthesia.

[17] Benumof J.L., Herway S.T. (2017). High end-tidal oxygen concentration can be a misleading sole indicator of the completeness of preoxygenation. Anesth Analg.

[18] Caputo N.D., Oliver M., West J.R., Hackett R., Sakles J.C. (2019). Use of end tidal oxygen monitoring to assess preoxygenation during rapid sequence intubation in the emergency department. Ann Emerg Med.

[19] Alkhouri H., Vassiliadis J., Murray M. (2017). Emergency airway management in Australian and New Zealand emergency departments: A multicentre descriptive study of 3710 emergency intubations. Emerg Med Australas.

[20] Edmark L., Kostova-Aherdan K., Enlund M., Hedenstierna G. (2003). Optimal oxygen concentration during induction of general anesthesia. Anesthesiology.

[21] Sakles J.C. (2017). Improving the safety of rapid sequence intubation in the emergency department. Ann Emerg Med.

[22] Oliver M., Caputo N.D., West J.R., Hackett R., Sakles J.C. (2020). Emergency physician use of end-tidal oxygen monitoring for rapidsequence intubation. J Am Coll Emerg Physicians Open.

[23] Stafford R.A., Benger J.R., Nolan J. (2008). Self-inflating bag or Mapleson C breathing system for emergency pre-oxygenation?. Emerg Med J.

[24] Robinson A.E., Pearson A.M., Bunting A.J. (2024). A Practical solution for preoxygenation in the prehospital setting: A nonrebreather mask with flush rate oxygen. Prehosp Emerg Care.

[25] Brown D.J., Carmichael J., Carroll S.M., April M.D. (2018). End-tidal oxygen saturation with nasal cannula during noninvasive positive pressure ventilation: A randomized crossover trial. J Emerg Med.

