# Supplementary material for: Assessment of Preoxygenation using Real-Time End-Tidal Oxygen Measurements Versus Single-Breath End-Tidal Oxygen Measurements in Healthy Volunteers
Source: J Am Coll Emerg Physicians Open. 2025 Feb 28;6(2):100079. doi: 10.1016/j.acepjo.2025.100079 (PMC11919592; doi:10.1016/j.acepjo.2025.100079)
Supplement: Supplementary Appendix 1-2 [file mmc1.docx]

**Appendix 1.** Formulas, estimated from our data, for Determining the Single-breath End-Tidal Oxygen Readings Based on Real-Time Nasal Cannula Readings for the Non-Invasive Ventilation (NIV) and Non-rebreather Mask (NRBM) Groups. Because the canonical link function of a beta regression is the logit link, the inverse logit link is required to convert back to the original units. NC values are entered on a 0 to 1 scale (e.g. 90% as 0.9). “e” is Euler’s number, a mathematical constant approximately equal to 2.718.


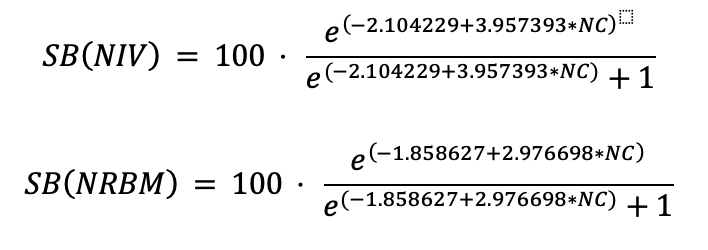

*SB*, single breath; *NC*, nasal cannula

**Appendix 2.** Kaplan-Meier curve depicting the rate at which participants reached their maximum end-tidal oxygen values. Dotted lines depict the median time to reach the maximum EtO_2_ value as a function of study arm.


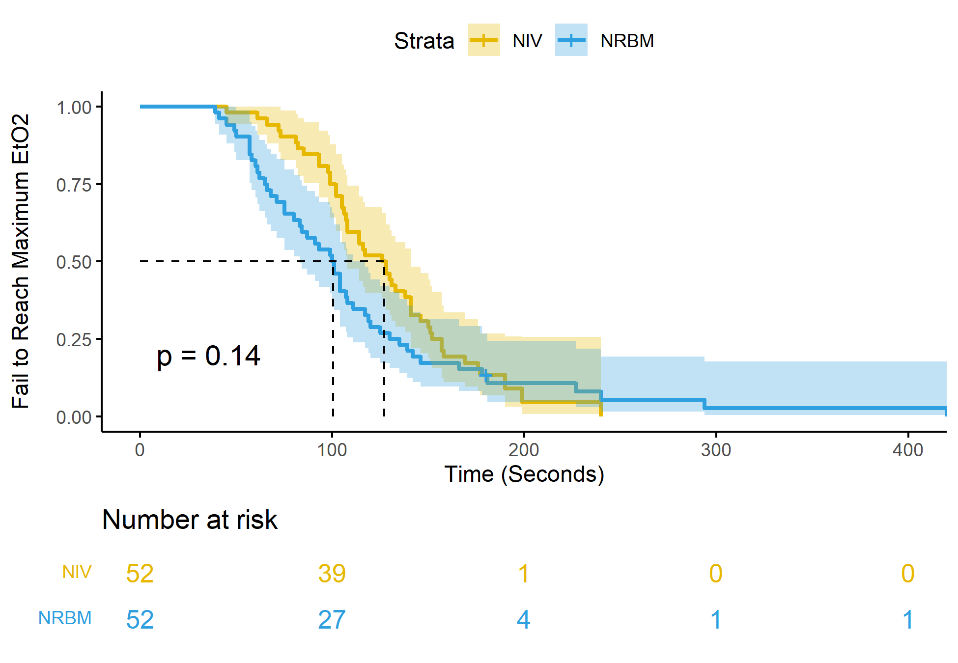


*NIV*, non-invasive ventilation study arm; *NRBM*, nonrebreather mask study arm.
